# ResSANet: Learning Geometric Information for Point Cloud Processing

**DOI:** 10.3390/s21093227

**Published:** 2021-05-06

**Authors:** Xiaojun Zhu, Zheng Zhang, Jian Ruan, Houde Liu, Hanxu Sun

**Affiliations:** 1 School of Automation, Beijing University of Posts and Telecommunications, Beijing 100876, China; zhu.xiaojun@sz.tsinghua.edu.cn (X.Z.); hxsun@bupt.edu.cn (H.S.); 2 Center for Artificial Intelligence and Robotics, Shenzhen International Graduate School, Tsinghua University, Shenzhen 518005, China; zheng-zh18@mails.tsinghua.edu.cn (Z.Z.); ruanjianoffice@126.com (J.R.)

**Keywords:** point-cloud processing, deep neural networks, machine learning, geometric primitives

## Abstract

Point clouds with rich local geometric information have potentially huge implications in several applications, especially in areas of robotic manipulation and autonomous driving. However, most point cloud processing methods cannot extract enough geometric features from a raw point cloud, which restricts the performance of their downstream tasks such as point cloud classification, shape retrieval and part segmentation. In this paper, the authors propose a new method where a convolution based on geometric primitives is adopted to accurately represent the elusive shape in the form of a point cloud to fully extract hidden geometric features. The key idea of the proposed approach is building a brand-new convolution net named ResSANet on the basis of geometric primitives to learn hierarchical geometry information. Two different modules are devised in our network, Res-SA and Res­SA­2, to achieve feature fusion at different levels in ResSANet. This work achieves classification accuracy up to 93.2% on the ModelNet40 dataset and the shape retrieval with an effect of 87.4%. The part segmentation experiment also achieves an accuracy of 83.3% (class mIoU) and 85.3% (instance mIoU) on ShapeNet dataset. It is worth mentioning that the number of parameters in this work is just 1.04 M while the network depth is minimal. Experimental results and comparisons with state-of-the-art methods demonstrate that our approach can achieve superior performance.

## 1. Introduction

With the rapid development of 3D acquisition technologies, high-precision point clouds are available. Point cloud representation preserves the original geometric information in a 3D space, for example, which enables point clouds that are versatile in many fields, including autonomous driving [1] and robotic manipulation [2]. However, point-cloud processing is an essentially intractable problem with several significant challenges [3,4], including the small scale of datasets, the high dimensionality and the unstructured and orderless nature of a 3D point cloud. Recently, many research areas are dominated by deep learning techniques, such as computer vision [5] and speech recognition [6]. While deep learning for 3D point clouds retains something of a gap in terms of practical applications, it has thus attracted increasing research attention. As mentioned, because of their orderless and unstructured nature, it is infeasible to apply standard convolutional neural networks (CNNs) directly to point clouds. In [3], the authors propose the pioneering work PointNet, which is able to work on irregular point clouds directly to learn per-point features using shared Multi-Layer Perceptron (MLP) and global features using a symmetrical pooling function. Based on PointNet, a series of pointwise MLP methods is proposed, such as PointNet++ [4], Frustum-PointNet [1], PointCNN [7], DGCNN [8], PointWeb [9]. Several publicly available datasets are also released, such as ModelNet40 [10] and ShapeNet [11].

However, pointwise features extracted by shared MLP cannot capture the local geometry in point clouds and the mutual interactions between points [3]. To capture a wider context for each point and obtain richer local hierarchy, several dedicated networks are introduced, including methods based on neighboring feature pooling [4], attention-based aggregation [12], and local-global feature concatenation [8,13,14]. Local-global feature fusion is an efficient method that reflects contextual information between a target and its surroundings.

One study [8] proposed EdgeConv, a novel simple operation that captures both the global shape structure and local neighborhood information and maintains permutation invariance. In particular, EdgeConv extracts edge features between a center point and its local k nearest neighborhood points. Another study [13] presented a learn-from-relation convolution operator, named RS-Conv, which uses 3D Euclidean distance as an intuitive description of low-level relation to encode geometric relation of points. Different from [8], [14] assumed that geometric information might be implicitly learned directly from the coordinates. They proposed Geo-Conv to explicitly model the geometric structure amongst points throughout the hierarchy of feature extraction, which is applied to each point in which the local spherical neighborhood is determined by a radius. Although it is effective, deficiencies still exist in the above-mentioned methods. EdgeConv [8] only considers the distance between points when constructing the neighborhood, and it ignores the direction of the vector, which leads to incomplete local geometric information. RS-Conv [13] just models the 3D Euclidean distances between center point and all its neighbors, which does not accurately describe the geometric information. Moreover, Geo-Conv [14] represented a geometric relationship between a point and its neighbors, which is explicitly modeled on six bases, while the local geometric information might be implicitly learned directly from an angle formed by the given point and its neighbors. 

To conclude, current methods are facing two following challenges for geometric modeling. First, these methods ignore the geometric information representations related to point sets accurately, especially contour information. Second, there is no further improvement of the information flow between layers in the networks by aggregating multi-level and multi-scale features repeatedly in most point cloud analysis pipelines.

**Figure 1:**
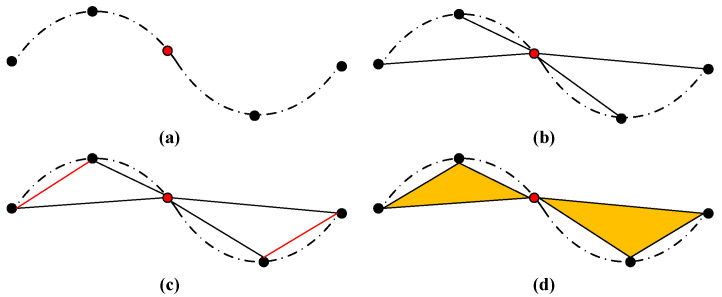
Different geometry representations on the continuous convex and concave surface of an object shape: (**a**) continuous convex and concave local surfaces; (**b**) taking the vectors between sampling points and KNN points; (**c**) adding the vectors between different KNN points; (**d**) multi-scale local geometric shape representation.

To address the above-mentioned problems, the authors propose a novel convolution-like operation based on geometric primitives to build our ResSANet. Here, the authors build a main network module—Res-SA module—referring to the conception of residual learning in Resnet [15] to accomplish a similar purpose, such as the Set Abstraction (SA) module in PointNet [1]. Firstly, different from DGCNN [8], in RS-Conv [13] and Geo-Conv [14], three different level features are explored, point-edge-face, as the bottom of Figure 1 shows. 

In Figure 1, the dotted lines indicate the outline of the object, the red points represent the sampling points, and the black points are the k-nearest neighbor (KNN) points of the corresponding sampling point. Old methods only consider the vectors between sampling points and KNN points like (b). However, only by taking some vectors between different KNN points like (c) into consideration can the network represents the object’s shape (like (d)) more accurately. In this way, multi-level features can be obtained, which is beneficial to understanding the point cloud accurately. Secondly, inspired by the deep residual learning in image recognition [15], in order to further improve the efficiency of information flow between different level features, the authors design a new skip connection mode by repeatedly aggregating multi-level and multi-scale features. Accordingly, two kinds of point-based skip connection modules are introduced, Res-SA and Res-SA-2, to obtain rich geometric information for point-cloud processing. As a result, ResSANet acquires various levels of local geometric information represented by geometric primitives to significantly improve work efficiency.

In order to help readers understand this paper, the primary contributions of this work are summarized as following:

1. A novel operation is presented based on geometric primitives for point clouds that could better capture local geometric features of point cloud while still maintaining permutation invariance;
2. Two point-based skip connection modules are devised in the network, Res-SA and Res-SA-2, which can fuse multi-level features to raise accuracy and efficiency in point-cloud processing;
3. The authors conduct extensive analyses and test the ResSANet. The results demonstrate they achieve state-of-the-art performance on challenging benchmark datasets, ModelNet40 [10] and ShapeNet [11], across three tasks, i.e., classification, shape retrieval and part segmentation.

## 2. Related Work

In this section, we briefly review existing deep learning methods for point-cloud processing.

### 2.1. Point-Cloud Processing Networks

**Projection-based networks.** These networks project point clouds into different representation modalities for feature learning, such as multi-view, volumetric representations. View-based methods usually first project a 3D object into multiple views, extract the corresponding view-wise features and then accomplish some specific tasks with the features (e.g., classification and segmentation). In this part, MVCNN [16], a pioneering work in terms of these methods, builds classifiers of 3D shapes from 2D image renderings of model shapes and combines max-pooling layers output and multi-view features into a global descriptor. However, a max-pooling operation may result in information loss. Several other methods, such as GVCNN [17], are proposed to improve the recognition accuracy. Volumetric-based methods always apply 3D Convolution Neural Network built on the volumetric representation of a point cloud. One study [18] first proposed a volumetric occupancy network named VoxNet to achieve a robust and fast object class detection for 3D point cloud. Another study [19] presented Voxelnet, which subdivides 3D space into equidistant voxels, and encoded the point cloud in each voxel into a united feature representation by a voxel feature-encoding layer. However, view-based methods lose some spatial information due to self-occlusions; therefore, volumetric-based methods always produce 3D grids, which are sparsely occupied in 3D space.

**Point-based networks.** According to the architecture used in the feature learning process of each point, point-based networks can be divided into Multi-Layer Perceptron (MLP)-based and convolution-based networks. As a pioneering work [3], proposed PointNet, which uses pointwise MLPs and aggregates global features utilizing symmetric functions to maintain permutation invariance. Since the local structural information of the point cloud is also an essential part of point-cloud processing, another work [4] presented a hierarchical network, PointNet++, to capture local geometric features from the neighborhood of each point and handle non-uniform sampling density problem. Due to the irregularity of a point cloud, a convolutional kernel for point cloud is hard to design. Current 3D convolution networks, for instance RSCNN [13] and Densepoint [20], define convolutional kernels on a continuous space, where the weights for neighboring points are related to the spatial distribution with respect to the center point. Other methods, such as PointCNN [7], Geo-Conv [14], W-CNN [21] and A-CNN [22], define convolutional kernels on regular grids where the weights for neighbors are related to the offsets with respect to the center point.

### 2.2. Deep Learning on Geometry

To exploit the local geometric structures, geometry-based networks represent each point in the point cloud as a vertex of a graph and then generate corresponding directed edges for the graph, and feature learning is performed in spatial space. One study [8] generated edge features that describe the geometric relations between each point and its neighbors, while the feature is determined by the center point coordinates and directed vectors pointing to the neighbors of each center point. Another study [23] represented that geometric structure of neighbor points by kernels and feature learning is based on kernel correlation. One group [24] proposed Rigorously Rotation-Invariant (RRI) module to captures rotation-invariant features for each point and constructed an unsupervised hierarchical clustering to learn the underlying geometric structure of point cloud. As a view-based 3D object classification method, another study [25] detailed the importance of geometric structures in the local shape and thus proposed a descriptor named Global Point Signature Plus (GPSPlus) module to capture more shape information. Besides, the work in [26] combines the coarse discrete grid structure with so-called continuous generalized Fisher vectors to represent the 3D point cloud and achieves impressive results. The work in [27] describes the design of a multi-level description of surfaces combined with the hierarchical decomposition of object shapes to represent the geometry. These works illustrate that the geometry representation of 3D shapes is important for point cloud understanding. Nevertheless, the authors in [28] expand the concept of DGCNN [8] to capture not only the intrinsic features of point cloud but also the extrinsic ones so that the network can learn geometry representations better. Similarly, an EdgeConv based feature fusion method is adopted by [29], in which an adaptive feature fusion module helps to learn both global and local features.

## 3. Approach

In this section, we first elaborate the convolution operator based on geometric primitives. Then, we show our network architectures used for point cloud classification and part segmentation. In both network architectures, Res-SA and Res-SA-2 modules are used. Thus, in the next two subsections, we explain the presented Res-SA and Res-SA-2 modules in detail, including their structure and function.

### 3.1. Geometric Primitives

Due to the irregular property, it is difficult to implement a classic convolution operator on a point cloud directly. We address this problem by adopting an efficient end-to-end permutation invariant convolution for point-cloud processing, which is based on geometric primitives. Consider a three-dimensional point cloud with n points and the points at the input level are represented by their 3D coordinates. The usual geometric-primitives-based convolution operator implemented on point cloud can be formulated as:

(1) $$F_{x_{i}}=\sigma (\alpha (\beta (x_{i})))$$

where $F_{x_{i}}$ is the high dimensioned feature vector extracted from the 3D coordinates of the *i*th point $x_{i}\in R^{3}$. $F_{x_{i}}$ can be permutation-invariant only when the first function β is shared over each point, and the second function α is symmetric (e.g., max) followed by a nonlinear activator function σ.

In order to make the extracted original point-cloud information more comprehensive, many researchers have explored the use of point [3] or edge [8] convolution. For convex or concave local surfaces, it is sufficient to use the vector relationship between the center point and any point in the sampling field to represent the local geometric information. However, for continuous convex and concave local surfaces as Figure 1a illustrates, only using the above-mentioned edge vectors to represent the local geometric information is inaccurate, as Figure 1b shows. Therefore, when it comes to represent the geometric information, we combined different level features, point-edge-face, as Figure 1c,d shows.

To conclude, we use $\left(x_{s}◇x_{k}◇x_{s}-x_{k}◇||x_{s}-x_{k}|{|}_{2}◇x_{k}-x_{k^{’}}◇||x_{k}-x_{k^{’}}|{|}_{2}\right)$ to replace the $x_{i}$ item in Equation (1) to represent the different level information of raw point clouds, where the center points $x_{s}$ and different neighborhood points $x_{k},x_{k^{’}}$ are also 3D positions in (x, y, z) form, ◇ is the concatenation operation and $||\cdot |{|}_{2}$ represent the Euclidean distance in 3D space. In such a combination, $\left(x_{s},x_{k}\right)$ denotes the point level and $\left(x_{s}-x_{k},||x_{s}-x_{k}|{|}_{2},x_{k}-x_{k^{’}},||x_{k}-x_{k^{’}}|{|}_{2}\right)$ indicates edge level. Besides, the vectors between sampling points $x_{s}$ and neighbor points $x_{k}$, as well as the vectors between different neighbor points $x_{k}$ and $x_{k^{’}}$ would form the the face-level information, which is also included in $\left(x_{s}-x_{k},||x_{s}-x_{k}|{|}_{2},x_{k}-x_{k^{’}},||x_{k}-x_{k^{’}}|{|}_{2}\right)$. Intuitively, this design could capture the multi-level feature of the raw point clouds.

### 3.2. ResSANet for Point-Cloud Processing

**Figure 2:**
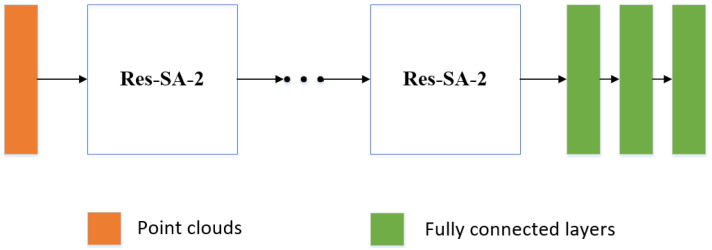
Classification Architecture. ResSANet applied in point cloud classification.

**Figure 3:**
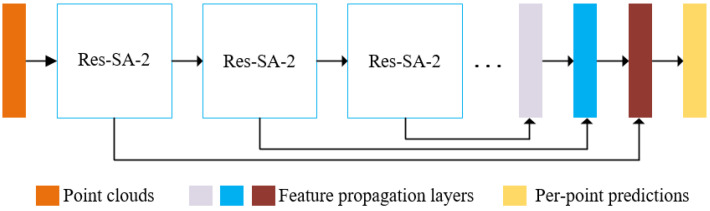
Part Segmentation Architecture. ResSANet applied in point cloud segmentation, in other words: per-point predictions.

In this work, ResSANet is adopted in point cloud classification and part segmentation tasks and both network structures are illustrated in Figure 2 and Figure 3, respectively. In each point-cloud processing task, Res-SA and Res-SA-2 modules are applied in each stage of the network to learn densely local geometric information. For classification, the final global representation is learned by two Res-SA-2 modules and three transition layers I, followed by three fully connected layers to achieve classification functions. For part segmentation, the representations learned by three Res-SA-2 modules (three transition layer II and six Res-SA modules) are upsampled by feature propagation layers [4] to generate per-point predictions.

### 3.3. Res-SA Module

In this part, our goal is to explain how Res-SA module learns densely local geometric information for point-cloud processing. Inspired by PointNet++ [4] and Resnet [15], this is different from classic CNN architecture in which the output feature $f_{n}$ of the *n*th layer is only learned from the output feature $f_{n-1}$ of its previous layer as:

(2) $$f_{n}=\phi (f_{n-1})$$

where ϕ(.) is one layer in classic CNN. As the network deepens, the more backward the layers are, the more difficult it is to comprehend the geometric feature information learned by the foremost layer, which leads to ineffectiveness while recognizing similar shapes.

To deal with non-uniform sampling density, [4] proposed multi-scale grouping, which captures multi-scale patterns to apply grouping layers with different scales and followed by corresponding small-scale PointNets [3] to extract features of each scale. Features at different scales are concatenated to form a multi-scale feature. In Pointnet++ [4], the set abstraction directly concatenates different levels after multi-scale grouping (MSG) or multi-resolution grouping (MRG). However, we argue that the operation o directly concatenating multi-scale features is unreasonable because different scale always means different semantic information for point clouds. To address this problem, inspired by Resnet [15], we propose a model called the Res-SA module in which we design a skip connection to further improve the information flow between adjacent layers and achieve feature fusion at different levels.

**Figure 4:**
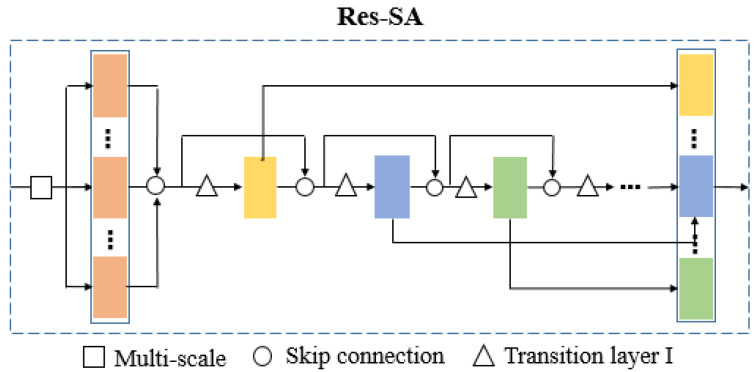
Res-SA. The diagram shows the proposed Res-SA architecture.

**Skip connection.** As Figure 4 illustrates, we first used the farthest sampling and grouping like [3] to extract low-level features from raw points, then performed a new skip connection mode by repeatedly aggregating multi-level and multi-scale features. The results of each scale grouping have been connected densely in MSG. For each scale feature layer in Res-SA, similar to [15], the outputs of two preceding layers are used as its input:

(3) $$f_{n}=\phi (\lambda (f_{n-2},f_{n-1}))$$

where $\left(f_{n-2},f_{n-1}\right)$ is the concatenation of the feature-maps outputted by layers n−1 and n−2, and ϕ(.) also represents one layer in classic CNN. And λ(.) is the transition layer I to keep the size of feature-maps after concatenated $\left(f_{n-2},f_{n-1}\right)$ the same as $f_{n-1}$.

**Transition layer I.** For ease of implementation, we need to keep the size of the feature maps constant. Thus, we design a pointwise transition layer that executes convolution and pooling. The pointwise transition layer used in our experiments consists of a batch normalization layer, a 1 × 1 convolutional layer followed by a max-pooling layer, an activation function layer and a dropout layer.

### 3.4. Res-SA-2 Module

**Figure 5:**
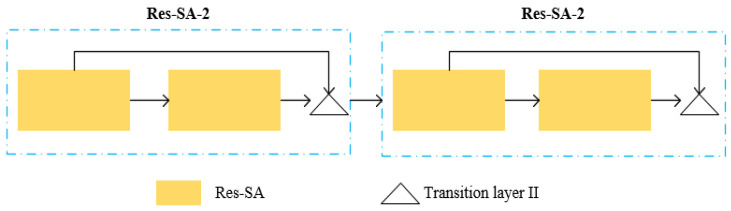
Res-SA-2. Architecture of our Res-SA-2 module is composed of several Res-SA modules. In this figure, we show a Res-SA-2 module consisting of two Res-SA modules.

Consider the relationship between one and another Res-SA module; we also define a new skip connectivity pattern, Res-SA-2:

(4) $$f_{n}=\phi (\Lambda (f_{n-2},f_{n-1}))$$

where ϕ(.) represents a neural network layer and Λ is the transition layer II. As Figure 5 demonstrates, Res-SA-2 module makes the connection between the Res-SA modules. The layers between two adjacent Res-SA modules are referred to as transition layer II in which convolution and pooling are applied to change feature map sizes.

**Transition layer II.** To avoid adding complexity as the network becomes deeper, we proposed a transition layer II, which consists of a batch normalization layer, an activation function layer, a 1 × 1 convolutional layer followed by a max-pooling layer.

## 4. Evaluation

### 4.1. Dataset and Implementation

We tested the proposed ResSANet on a ModelNet40 dataset [10], which included CAD models of 40 categories, and we used the official split with 9843 shapes for training and 2468 for testing in classification and shape retrieval tasks. We also trained a part segmentation network on the ShapeNet part benchmark [30], which contains 16,881 shapes with 16 categories and is labeled in 50 parts in total. All experiments ran on a desktop computer running Ubuntu 16.04 with a 3.60 GHz Intel Core i9-9900K CPU and an NVIDIA 2080Ti.

### 4.2. Classification

In the training stage, we uniformly sampled 1024 input points from 3D Euclidean spaces. While in the testing stage, we conducted 10 voting tests with random scaling and obtained the average predictions similar to PointNet [3] and PointNet++ [4]. The quantitative comparisons with other point-based methods are summarized in Table 1, which shows that our proposed approach outperforms all the xyz input methods. Note that in Table 1, n means extra normal information.

**Figure 6:**
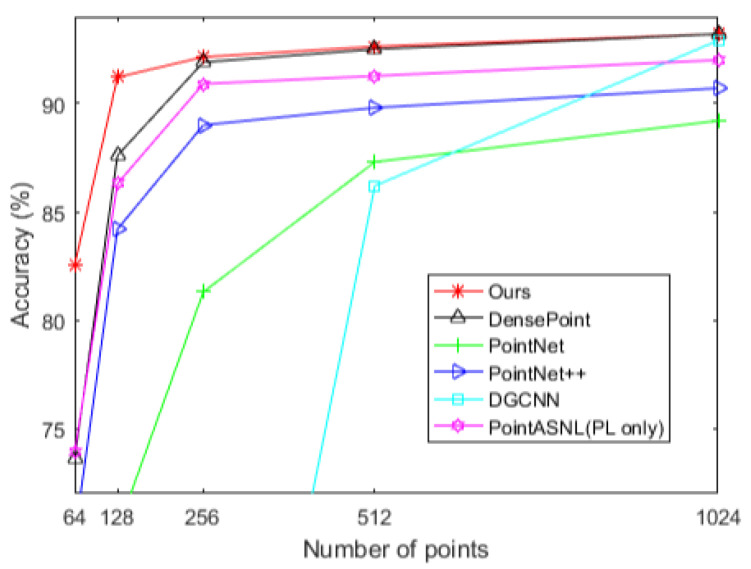
Robustness. The comparisons of testing results with sparser points.

Surprisingly, the work achieved an accuracy of 93.2%, which could be comparable to most of the state-of-the-art methods with additional normal information or multi-view with quite dense point inputs. We also tested the robustness of ResSANet on different sampling densities by using sparser points of number 64, 128, 256, 512 and 1024 as the input to a model trained with 1024 points. In the training phase, the input point cloud was randomly discarded with a probability range from 0 to *p* (0 < *p* < 1) to enhance the network’s robustness to the point cloud scale. In the actual test phase, for a specific number of points, a fixed probability was used to discard and got the classification accuracy of the points. We compared the proposed ResSANet against PointNet [3], PointNet++ [4], Densepoint [20] and PointASNL [42], and the results are shown in Figure 6. Intuitively, our work can obtain the best accuracy in very sparse point clouds (e.g., 64 and 128 points).

**Figure 7:**
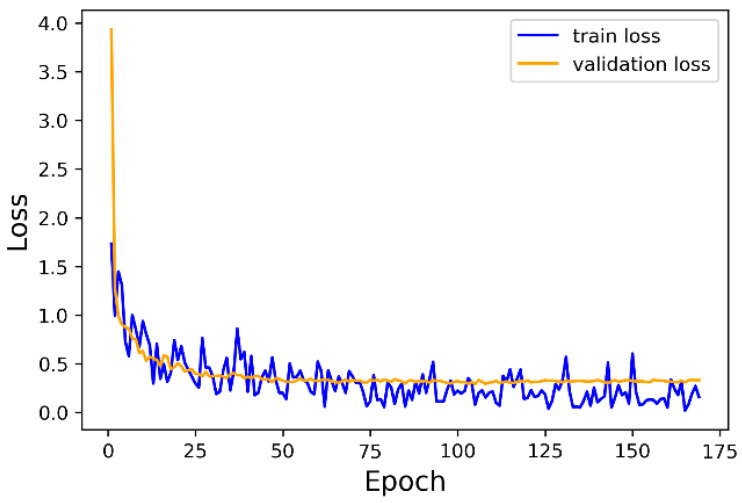
Training and validation loss curve during training the network for object classification task.

To illustrate that the high accuracy of classification does not benefit from some training tricks such as overfitting, we show our training plot in Figure 7. In the classification experiment, we set several random cross-validation groups during training. Once an epoch is finished, the network parameters are fed into training group and cross-validation group separately. From Figure 7, we can see that validation loss is decreasing together with train loss, including when the network has already been stable after around 100 epochs.

### 4.3. Shape Retrieval

**Figure 8:**
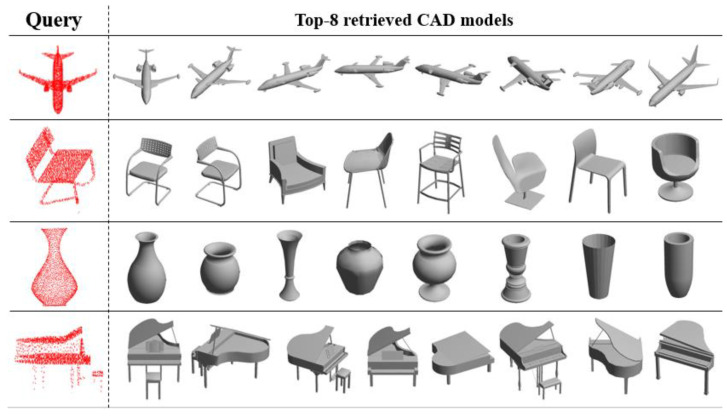
Shape Retrieval Experiment. Results on ModelNet40 benchmark. From top to bottom, we show the top eight most similar retrieval results on an airplane, chair, bottle and piano.

The previous classification network can be easily extended to the task of 3D shape retrieval by applying the outputs of the fully connected layer as the feature vector. The similarity between the query shape and the shape library candidates can be computed as their feature vector L2 distances and mean Average Precision (mAP). As Table 2 shows, our ResSANet is the state-of-art point-based methods, which outperforms PointNet [3] by 15.7%. We have also compared some advanced methods based on 2D images (shown in Table 2) and achieved comparable results to Triplet [44], which greatly benefited from 2D image CNN and pre-training with ImageNet [5] datasets. Figure 8 shows some of the retrieval results on ModelNet40 dataset on an airplane, chair, bottle and piano, which identifies the validity of our ResSANet on a shape retrieval task.

### 4.4. Part Segmentation

**Figure 9:**
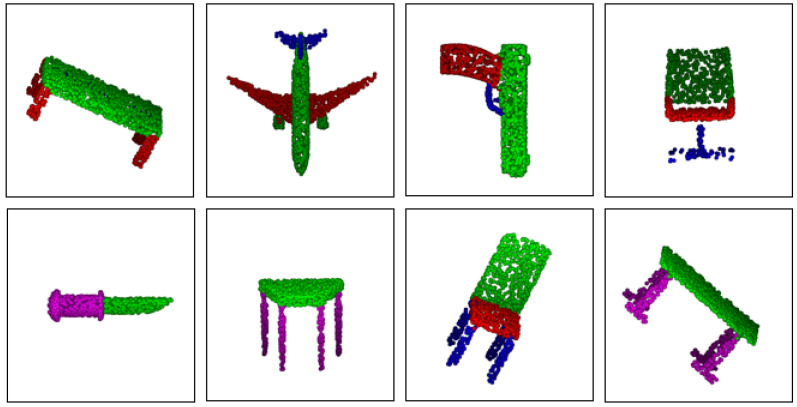
Part Segmentation Experiment. Results on ShapeNet part benchmark. Different colors imply different parts on a given shape.

We deliberately reformulated the part segmentation problem as a per-point classification task, as illustrated in Figure 3, and randomly picked 2048 points as the input and concatenated the one-hot encoding of the object label to the last feature layer of the segmentation network. In the testing stage, we also apply voting with 10 tests using random scaling. Besides the standard Inter-over-Union (IoU) score for each category, two types of mean IoU (mIoU) that are averaged across all classes and all instances, respectively, are also calculated. The quantitative comparisons with the state-of-the-art point-based methods are summarized in Table 3, and intuitively, the ResSANet outperforms all the point-input methods. Moreover, it significantly surpasses PointNet [3] with 2.9% increase in class mIoU and 1.6% increase in instance mIoU, respectively, which proves the validity of this method. Figure 9 shows some of the part segmentation results on a ShapeNet dataset. It can be said that these objects are segmented into accurate parts intuitively.

### 4.5. Model Complexity

We define our network depth L as the number of Res-SA modules in each Res-SA-2 module. To explore the impact of depth, we tested our classification network with depth L being 1, 2 and 3 on ModelNet40. The proposed ResSANet cloud achieved the best result of 93.2% in classification experiments when depth L is 2. We evaluated the amount of model parameters and floating-point operation per second (FLOPs) of several point cloud-based networks in the task of ModelNet40 classification, as shown in Table 4. Note that the ResSANet is quite competitive, and it can be the most efficient one with the network depth L being 1. Besides, the last three lines in Table 4 summarizes the impact of L on the proposed ResSANet. The reasons why our network is lightweight are summarized as follows: In transition layers, we adopt pointwise convolution (1×1 convolutional layer), which is known as a valid way to reduce the number of parameters. We do not introduce any extra parameters in the proposed skip connection operation. Thanks to the hierarchical architecture, we can simply reduce the depth of our network to achieve a balance between performance and complexity.

## 5. Conclusions

In this work, a novel architecture named ResSANet is proposed, to densely learn local geometric information for point-cloud processing. ResSANet captures local geometric information by geometric primitives and represents the relevant efficient generalized convolution operator. Based on this convolution operator, ResSANet could extract multi-level and multi-scale features. Accordingly, ResSANet further improves the information flow in the architecture, improving its efficiency significantly for learning geometric information. The experiments comparing ResSANet against other state-of-art methods were performed on challenging benchmark datasets across three tasks, i.e., classification, shape retrieval and part segmentation, which thoroughly validated the outstanding performance of ResSANet and the remarkable robustness to quite a sparse point cloud. 

However, one drawback of this work is that some parameters exist that require adjustment during training. The overall performance of the network is closely related to these parameters, such as the sampling radius, the number of layers, the number of channels and so on. Therefore, one of the work directions in the next stage that needs improvement is to fuse these parameters into the network to improve the network’s stability and trainability.

## Figures and Tables

**Table 1:** Classification results on a ModelNet40 dataset.

Algorithm	Input	Points	Accuracy
ClusterNet [24]	xyz	1024	87.1%
PointNet [3]	xyz	1024	89.2%
SCN [31]	xyz	1024	90.0%
Kd-Net [32]	xyz	1024	90.6%
PointNet++ [4]	xyz	1024	90.7%
MCConv [33]	xyz	1024	90.9%
KCNet [23]	xyz	1024	91.0%
MRTNet [34]	xyz	1024	91.2%
Spec-GCN [35]	xyz	1024	91.5%
W-CNN [21]	xyz	1024	92.0%
DGCNN [8]	xyz	1024	92.2%
PointCNN [7]	xyz	1024	92.2%
PCNN [36]	xyz	1024	92.3%
PointWeb [9]	xyz	1024	92.3%
Point2Sequence [37]	xyz	1024	92.6%
A-CNN [22]	xyz	1024	92.6%
LDGCNN [38]	xyz	1024	92.9%
PointASNL [39]	xyz	1024	92.9%
Ours	xyz	1024	93.2%
FoldingNet [40]	xyz	2048	88.4%
SO-Net [41]	xyz	2048	90.9%
Spec-GCN [35]	xyz + n	1024	91.8%
Pointconv [42]	xyz + n	1024	92.5%
Geo-CNN [14]	xyz + n	1024	93.4%
SpiderCNN [43]	xyz + n	5000	92.4%
LDGCNN [38]	xyz + n	5000	92.9%
MLVCNN [44]	xyz + n	5000	92.9%
SO-Net [41]	xyz + n	5000	93.4%
PVNet [45]	xyz + n	1024	93.2%

**Table 2:** Shape retrieval results (mAP, %) on ModelNet40 Dataset.

Modality	Algorithm	Points/Views	mAP
points	PointNet [3]	1 k	70.5
points	PointCNN [7]	1 k	83.8
points	DGCNN [8]	1 k	85.3
points	Ours	1 k	87.4
Images	3D ShapeNet [10]	-	49.2
Images	MVCNN [16]	12	80.2
Images	GIFT [46]	12	81.9
Images	GVCNN [17]	12	85.7
Images	PANORAMA-ENN [47]	-	86.3
Images	Triplet [48]	12	88.0
Images	SeqViews [49]	12	89.1

**Table 3:** Part-segmentation results (%) on ShapeNet part benchmark.

Algorithm	Ours	PointNet	KCNet	DGCNN	PCNN
points	2048	2048	2048	2048	2048
Class	83.3	80.4	82.2	82.3	81.8
Instance	85.3	83.7	84.7	85.1	85.1
airplane	82.0	83.4	82.8	84.2	82.4
bag	85.7	78.7	81.5	83.7	80.1
cap	87.2	82.5	86.4	84.4	85.5
car	78.1	74.9	77.6	77.1	79.5
chair	90.5	89.6	90.3	90.9	90.8
earphone	78.9	73.0	76.8	78.5	73.2
guitar	91.2	91.5	91.0	91.5	91.3
knife	86.8	85.9	87.2	87.3	86.0
lamp	85.2	80.8	84.5	82.9	85.0
laptop	95.6	95.3	95.5	96.0	95.7
motorbike	71.9	65.2	69.2	67.8	73.2
mug	94.5	93.0	94.4	93.3	94.8
pistol	83.1	81.2	81.6	82.6	83.3
rocket	61.4	57.9	60.1	59.7	51.0
skateboard	77.4	72.8	75.2	75.5	75.0
table	82.6	80.6	81.3	82.0	81.8

**Table 4:** The comparisons of model complexity on ModelNet40 Dataset.

Algorithm	Params	FLOPs
PCNN [7]	8.20 M	294 M
PointNet [3]	3.50 M	440 M
RGCNN [50]	2.24 M	750 M
SpecGCN [7]	2.05 M	1112 M
DGCNN [7]	1.84 M	2767 M
PointNet++ [7]	1.48 M	1684 M
RSCNN [13]	1.41 M	295 M
Ours (L = 3)	1.59 M	450 M
Ours (L = 2)	1.31 M	409 M
Ours (L = 1)	1.04 M	286 M

## Data Availability

The data presented in this study are available on request from the corresponding author.
